# Mediating effect of adiponectin between free fatty acid and tumor necrosis factor-α in patients with diabetes

**DOI:** 10.1038/s41387-024-00302-5

**Published:** 2024-06-17

**Authors:** Zhang Xia, Shulong Shi, Xiaoqing Ma, Feng Li, Xinya Li, Herbert Y. Gaisano, Mingyang Zhao, Yuhao Li, Yan He, Jiajia Jiang

**Affiliations:** 1 Department of Epidemiology and Health Statistics, School of Public Health, Capital Medical University, Beijing, China; 2 Beijing Municipal Key Laboratory of Clinical Epidemiology, Beijing, China; 3 Department of Endocrinology, Jining No. 1 People’s Hospital, Jining, Shandong China; 4 Institute for Chronic Disease Management, Jining No. 1 People’s Hospital, Jining, Shandong China; 5 Departments of Medicine and Physiology, University of Toronto, Toronto, ON Canada; 6 Postdoctoral of Shandong University of Traditional Chinese Medicine, Jinan, Shandong China

**Keywords:** Type 2 diabetes, Type 2 diabetes

## Abstract

**Background/Objectives:**

Increased free fatty acid (FFA) promotes adiponectin secretion in healthy subjects and induces inflammation in diabetes. Given the potential pro-inflammatory role of adiponectin in “adiponectin paradox”, we performed this study in patients with type 2 diabetes mellitus (T2DM) to assess the association of FFA with adiponectin and to investigate whether adiponectin mediates FFA-related inflammation.

**Methods:**

This cross-sectional study consisted of adult patients with T2DM. FFA, adiponectin, and tumor necrosis factor-α (TNF-α) were assayed from fasting venous blood after overnight fasting for at least 8 h. Multivariable linear regression analysis and restricted cubic splines (RCS) analysis were performed to identify the association between FFA and adiponectin. Mediation analysis was performed to determine the mediating effect of adiponectin on the association between FFA and TNF-α.

**Results:**

This study included 495 participants, with 332 males (67.1%) and a mean age of 47.0 ± 11.2 years. FFA was positively associated with adiponectin (*b* = 0.126, 95%CI: 0.036–0.215, *P* = 0.006) and was the main contributor to the increase of adiponectin (standardized *b* = 0.141). The RCS analysis demonstrated that adiponectin increased with FFA when FFA was less than 0.7 mmol/L but did not further increase thereafter (*P*_overall_ < 0.001 and *P*_non-linear_ < 0.001). In addition, adiponectin mediated the association between FFA and TNF-α. The mediating effect was 0.08 (95%CI: 0.03–0.13, *P* = 0.003) and the mediating effect percentage was 26.8% (95%CI: 4.5–49.2, *P* = 0.02).

**Conclusions:**

In patients with T2DM, FFA was positively associated with adiponectin when FFA was less than 0.7 mmol/L. Elevated adiponectin mediated FFA-related inflammation. This study may provide insights into the pro-inflammatory effect of adiponectin in T2DM.

## Introduction

Adiponectin as a pleiotropic adipokine is thought to increase insulin sensitivity and reduce inflammation, thereby protecting the heart, vasculature, kidney, and colon from injury during stress in the general population [1]. However, the “adiponectin paradox” has been observed in diabetes and other clinical conditions and increased adiponectin was associated with higher risk for cardiovascular disease and all-cause mortality [2, 3]. Although the biology underlying this paradox is unknown, possible explanations include adiponectin resistance, the confounding role of natriuretic peptides, and reduced excretion of adiponectin from the kidneys [2]. Notably, a recently proposed explanation is that adiponectin might have pro-inflammatory rather than anti-inflammatory effects in people living with chronic conditions [2]. Nonetheless, clinical data supporting this explanation are still limited.

Dysmetabolism of free fatty acid (FFA) is the pathological basis for the development of type 2 diabetes mellitus (T2DM) [4]. Notably, clinical trials have shown that acute change in FFA concentrations was positively associated with the subsequent alteration in adiponectin concentrations [5, 6], indicating that increased FFA might promote adiponectin secretion. Additionally, a few studies have found that adiponectin appears to have pro-inflammatory effects under inflammatory conditions, and mechanisms involve macrophage polarization, T cell differentiation, the activation of intracellular protein kinases pathway, and nuclear factor-kappa B pathway, etc [7–10]. Given the well-known link between FFA and inflammation [11], we proposed a hypothesis that FFA may stimulate adiponectin secretion and adiponectin in turn mediate FFA-related inflammatory reaction in T2DM.

Proving the current hypothesis may advance the understanding of the biological function of adiponectin and the phenomenon of “adiponectin paradox” in people with diabetes. Nevertheless, two issues should be addressed before verification of this hypothesis. First, the association between FFA and adiponectin was investigated in healthy subjects [5, 6]. It remains unclear whether FFA is positively associated with adiponectin in T2DM. Second, little is known about the mediating effect of adiponectin between FFA and inflammatory cytokine. We therefore performed this cross-sectional study in adult patients with T2DM, aiming to assess the association between FFA and adiponectin and then to investigate whether adiponectin mediates FFA-related inflammatory reaction.

## Materials and methods

### Study design and participants

This was a cross-sectional study. We included 495 patients, who were admitted at the National Metabolic Management Center of Jining First People’s Hospital Affiliated to Shandong First Medical University from May 2020 to April 2022. All participants included in this study were adults (age ≥18 years) and diagnosed with T2DM. The study protocol was approved by the Committee on Human Research of the Jining NO.1 People’s Hospital (2021 Ethical Approval No. 025). All participants provided written informed consent. This study followed the Strengthening the Reporting of Observational Studies in Epidemiology reporting guideline and was conducted in accordance with the declaration of Helsinki.

### Clinical and laboratory measurements

Demographics, medical history, and medication use were collected using a standardized questionnaire through face-to-face interviews. Body height and weight were measured using height and weight meters (OMRON HNH-318; OMRON Corporation, Shenzhen, China). Blood pressure was measured using an electronic blood pressure meter (OMRON HBP-1100U; OMRON Corporation, Dalian, China) in a seated position after 5 min of rest.

The venous blood sample was drawn in the morning after overnight fasting for at least 8 h to measure the following laboratory indices. Triglyceride (TG), total cholesterol (TC), high-density lipoprotein cholesterol (HDL-C), low-density lipoprotein cholesterol (LDL-C), alanine aminotransferase (ALT), aspartate aminotransferase (AST), and serum creatinine were assayed enzymatically by an automatic biochemistry analyzer (AU5831, Beckman Coulter, USA). Plasma FFA and adiponectin were assayed by an automatic biochemistry analyzer (BS-2000M, Mindray, China), with ACS-ACOD method for FFA and immunoturbidimetry method for adiponectin. Tumor necrosis factor-α (TNF-α) was assayed using chemiluminescence method by an automatic chemiluminescence immunoanalyzer (IMMULITE1000, Siemens, USA). Glycosylated hemoglobin (HbA1c) was assayed by a hemoglobin analyzer (Bio-Rad D-10) using high-pressure liquid chromatography.

A standardized steamed bread meal test was performed in the morning after overnight fasting and the discontinuation of medication that would affect the test. The standard steamed bread meal is made of 100 g flour, which contains carbohydrates approximately equivalent to 75 g glucose, and has similar experiment results as the oral glucose tolerance test [12, 13]. The venous blood sample was drawn to measure blood glucose, insulin, and C-peptide at fasting, 60 min, 120 min, and 180 min following the ingestion of 100-g flour. Insulin and C-peptide were assayed by an automatic electrochemiluminescence analyzer (Cobas e801, Roche Diagnostics, Mannheim, Germany). Blood glucose was assayed enzymatically by an automatic biochemistry analyzer (AU5831, Beckman Coulter, USA).

### Index calculation

Body mass index (BMI) was calculated as weight (kg) divided by height (m) squared.

The estimated glomerular filtration rate (eGFR) was calculated by the Chronic Kidney Disease-Epidemiology Collaboration equation [14]. Homeostatic model assessment of insulin sensitivity (HOMA2S) and homeostatic model assessment of beta-cell function (HOMA2B) were calculated by the HOMA2 model using fasting blood glucose (FBG) (mmol/L) and fasting C-peptide (nmol/L) [15].

### Definitions

T2DM was defined as FBG ≥ 7.0 mmol/L, 2-hour blood glucose from the oral glucose tolerance test ≥11.1 mmol/L, HbA1c ≥ 6.5%, or self-reported physician-diagnosed diabetes [16]. Hypertension was defined as systolic blood pressure ≥140 mmHg, diastolic blood pressure ≥90 mmHg, any use of antihypertensive drugs, or self-reported history of hypertension [17]. Dyslipidemia was defined as TC ≥ 6.2 mmol/L, TG ≥ 2.3 mmol/L, HDL-C < 1.0 mmol/L, LDL-C ≥ 4.1 mmol/L, any use of lipid-lowering drugs, or self-reported history of dyslipidemia [18]. Chronic kidney disease was defined as having eGFR <60 mL/min/1.73 m^2^ [19]. Coronary heart disease and stroke were confirmed by self-reporting medical history.

### Statistical analysis

Continuous variables with normal distribution were described as mean ± standard deviation. Those with skewed distribution were described as median (P25–P75). Categorical variables were described as numbers and percentages. The distribution of adiponectin among FFA groups was visualized using the box plot and the trend of adiponectin levels was analyzed using the univariable linear regression model.

To identify the factors associated with adiponectin, we first performed the univariable linear regression analysis to screen potential influence factors. Screened factors included age, sex, duration of diabetes, BMI, TC, TG, LDL-C, HDL-C, FFA, HbA1c, FBG, fasting insulin, ALT, AST, current insulin therapy, current oral hypoglycemic agents therapy, and current lipid-lowering therapy. Notably, adiponectin, duration of diabetes, TG, HDL-C, FFA, ALT, AST, and fasting insulin were log-transformed due to skewness distribution. Then, the factors with *P* < 0.10 in the univariable model were selected and included in the multivariable linear regression model to compare their contributions to adiponectin.

To assess the dose-response relationship between FFA and adiponectin, we performed the restricted cubic splines (RCS) analysis. We set three knots at 5th, 50th, 95th percentiles of FFA and set 0.7 mmol/L of FFA as a reference. To avoid reverse causality, we repeated the RCS analysis with adiponectin as the independent variable and FFA as the dependent variable. *P*_overall_ < 0.05 and *P*_non-linear_ < 0.05 indicate a nonlinear association.

To determine whether adiponectin mediated FFA-related deleterious effects, we performed the mediation analysis. In the mediation model, we used FFA as the independent variable, adiponectin as the mediating variable, and TNF-α, HOMA2S, HOMA2B, HbA1c, dyslipidemia, hypertension, coronary heart disease, stroke, chronic kidney disease as the dependent variables. We estimated the mediating effect and the mediating effect percentage of adiponectin after adjustment for potential confounders. We further performed subgroup analyses in participants with different ages, sexes, HbA1c levels, and conditions of current insulin therapy.

All statistical analyses were conducted using SAS version 9.4 (SAS Institute Inc, Cary, NC). A two-tailed *P* < 0.05 was considered statistically significant.

## Results

### Characteristics of the participants

This study included 495 adult patients with T2DM, with 332 males (67.1%) and a mean age of 47.0 ± 11.2 years. The median duration of diabetes was 2.9 (0.1–8.2) years and the mean HbA1c was 9.4 ± 2.2% (79.5 ± 24.6 mmol/mol). In addition, the median values for FFA and adiponectin were 0.4 (0.3–0.7) mmol/L and 7.2 (5.0–10.7) μg/mL, respectively. Of participants, 133 (26.9%) underwent insulin therapy and 451 (91.1%) underwent oral hypoglycemic agents treatment (Table 1).

**Table 1:** Demographic and clinical characteristics of participants.

Characteristics	Value
*N*	495
Age, mean ± SD, year	47.0 ± 11.2
Male sex, *n* (%)	332 (67.1)
Duration of diabetes, median (P25–P75), year	2.9 (0.1–8.2)
Body mass index, mean ± SD, kg/m^2^	26.4 ± 4.1
Systolic blood pressure, mean ± SD, mmHg	129.5 ± 17.0
Diastolic blood pressure, mean ± SD, mmHg	80.4 ± 11.1
HbA1c, mean ± SD, %	9.4 ± 2.2
HbA1c, mean ± SD, mmol/mol	79.5 ± 24.6
Triglyceride, median (P25–P75), mmol/L	1.6 (1.1–2.5)
Total cholesterol, mean ± SD, mmol/L	4.7 ± 1.2
High-density lipoprotein cholesterol, median (P25–P75), mmol/L	1.0 (0.8–1.2)
Low-density lipoprotein cholesterol, mean ± SD, mmol/L	2.7 ± 0.9
Free fatty acid, median (P25–P75), mmol/L	0.4 (0.3–0.7)
Alanine transaminase, median (P25–P75), U/L	19.1 (13.7–29.7)
Aspartate aminotransferase, median (P25–P75), U/L	16.0 (12.8–21.5)
Adiponectin, median (P25–P75), μg/mL	7.2 (5.0–10.7)
Tumor necrosis factor-α, median (P25–P75), pg/mL	331.0 (214.0–491.0)
Hypertension, *n* (%)	229 (46.3)
Dyslipidemia, *n* (%)	348 (70.3)
Current insulin therapy, *n* (%)	133 (26.9)
Current oral hypoglycemic agents treatment, *n* (%)	451 (91.1)
Current angiotensin receptor blockers therapy, *n* (%)	90 (18.2)
Current angiotensin-converting enzyme inhibitor therapy, *n* (%)	11 (2.2)
Current statins therapy, *n* (%)	193 (39.0)

*HbA1c* glycosylated hemoglobin, *SD* standard deviation.

### Association between FFA and adiponectin

**Fig. 1:**
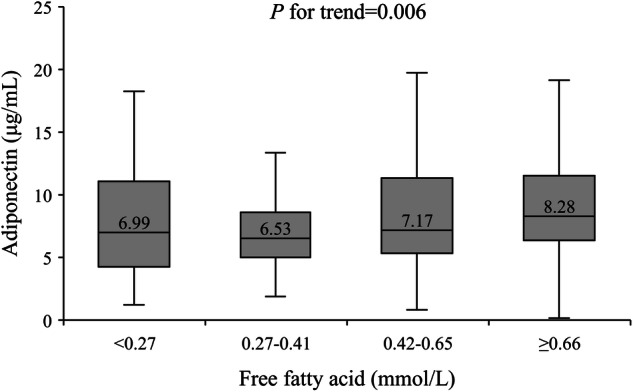
Adiponectin distribution in quartile groups of free fatty acid. Data in the box plot were median values of adiponectin. The sample sizes for free fatty acid groups of <0.27 mmol/L, 0.27–0.41 mmol/L, 0.42-0.65 mmol/L, and ≥0.66 mmol/L were 117, 129, 125, and 124, respectively.

After categorizing FFA into four groups (<0.27 mmol/L, 0.27-0.41 mmol/L, 0.42-0.65 mmol/L, and ≥0.66 mmol/L) based on its quartiles, the corresponding median levels of adiponectin were 6.99 μg/mL, 6.53 μg/mL, 7.17 μg/mL, and 8.28 μg/mL, respectively. There was an increasing trend in adiponectin levels from the lowest quartile group to the highest quartile group (*P* for trend = 0.006) (Fig. 1).

To identify the factors associated with adiponectin, we performed linear regression analyses in factors including age, sex, duration of diabetes, BMI, lipid profiles, FFA, HbA1c, FBG, fasting insulin, ALT, AST, antidiabetic therapy, and lipid-lowering therapy. We found that logHDL-C (*b* = 0.170, 95%CI: 0.005–0.334, *P* = 0.04) and logFFA (*b* = 0.126, 95%CI: 0.036–0.215, *P* = 0.006) were positively associated with log-transformed adiponectin. Notably, higher FFA was the main contributor to the increase of adiponectin (standardized *b* = 0.141). However, logTG (*b* = -0.144, 95%CI: −0.240 to −0.048, *P* = 0.003) and logALT (*b* = −0.238, 95%CI: −0.404 to -0.072, *P* = 0.005) were negatively associated with log-transformed adiponectin (Supplementary Table S1).

### Dose-response relationship between FFA and adiponectin

**Fig. 2:**
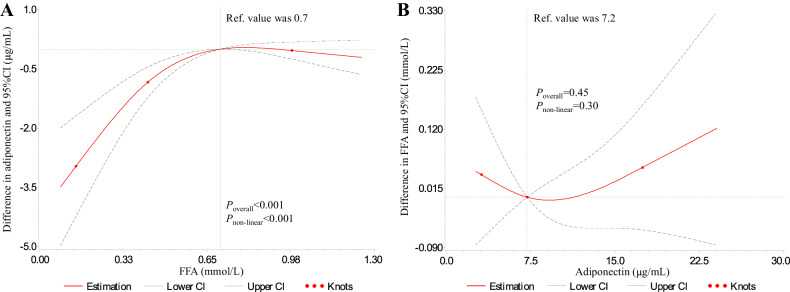
Dose-response relationship between FFA and adiponectin. **A** The relationship of FFA with adiponectin; **B** the relationship of adiponectin with FFA. *FFA* free fatty acid. Two models had the same sample size of 479 and both were adjusted for age, sex, duration of diabetes, body mass index, estimated glomerular filtration rate, alanine transaminase, current insulin therapy, current oral hypoglycemic agents treatment, and current statins therapy. To minimize the effect of extreme values of the independent variable on the proportion of figure, we drew above figure with an independent variable ranging from the 2.5th to 97.5th percentiles.

To identify the dose-response relationship between FFA and adiponectin, we performed the RCS analysis. We found that FFA was inverted J-shaped associated with adiponectin after adjustment for potential confounders (*P*_overall_ < 0.001 and *P*_non-linear_ < 0.001). Specifically, adiponectin significantly increased when FFA increased from low levels to 0.7 mmol/L. Then adiponectin had a decline trend when FFA was more than 0.7 mmol/L but this trend was not statistically significant due to a large confidence interval (Fig. 2A). To avoid reverse causality, we repeated the RCS analysis using adiponectin as the independent variable and FFA as the dependent variable. We found that adiponectin was not associated with FFA after adjustment for the same variables (*P*_overall_ = 0.45 and *P*_non-linear_ = 0.30) (Fig. 2B).

### The mediating effect of adiponectin on the association between FFA and TNF-α

The above findings demonstrated that increased FFA may promote adiponectin secretion in T2DM. Given the pleiotropic effect of adiponectin, we performed mediation analyses to identify the mediating effect of adiponectin on the association between FFA and FFA-related outcomes. Although the analyses were performed on the association of FFA with TNF-α, HOMA2S, HOMA2B, HbA1c, dyslipidemia, hypertension, coronary heart disease, stroke, and chronic kidney disease, we found that adiponectin only mediated the association between FFA and TNF-α. The corresponding mediating effect was 0.08 (95%CI: 0.03–0.13, *P* = 0.003) and the mediating effect percentage was 26.8% (95%CI: 4.5–49.2, *P* = 0.02) (Table 2).

**Table 2:** Mediating effect of adiponectin on the association between FFA and FFA-related outcomes.

Outcome	*N*	Events	Mediating effect of adiponectin		Mediating effect percentage of adiponectin	
			Coefficient (95% CI)	*P* value	Coefficient (95% CI)	*P* value
Continuous variable						
TNF-α^a^	475	–	0.08 (0.03–0.13)	0.003	26.8 (4.5–49.2)	0.02
HOMA2S^b^	366	–	0.002 (−0.008–0.01)	0.64	−14.3 (−104.1–75.6)	0.76
HOMA2B^b^	366	–	−0.004 (−0.01–0.01)	0.39	−12.2 (−49.6–25.3)	0.52
HbA1c^c^	475	–	0.04 (−0.01–0.09)	0.09	−69.8 (−393.6–254.0)	0.67
Binary variable						
Dyslipidemia^d^	478	332	−0.13 (−0.26–0.003)	0.06	−17.8 (−37.5–2.0)	0.08
Hypertension^a^	475	219	−0.01 (−0.09–0.06)	0.73	−3.4 (−22.2–15.5)	0.73
Coronary heart disease^e^	478	51	−0.01 (−0.06–0.03)	0.57	5.9 (−17.1–28.9)	0.62
Stroke^e^	478	34	0.05 (−0.07–0.17)	0.40	12.0 (−20.6–44.5)	0.47
Chronic kidney disease^f^	487	13	0.09 (−0.11–0.30)	0.36	24.0 (−47.5–95.6)	0.51

*FFA* free fatty acid, *TNF-α* tumor necrosis factor-α, *HbA1c* glycosylated hemoglobin, *HOMA2S* homeostatic model assessment of insulin sensitivity, *HOMA2B* homeostatic model assessment of beta-cell function, *eGFR* estimated glomerular filtration rate, *TG* triglyceride, *TC* total cholesterol, *HDL-C* high-density lipoprotein cholesterol, *LDL-C* low-density lipoprotein cholesterol.

FFA, adiponectin, TNF-α, HOMA2S, and HOMA2B were log-transformed due to skewness distribution.

Covariates included age, sex, duration of diabetes, body mass index, alanine transaminase, current insulin therapy, current oral hypoglycemic agents treatment, and current statins therapy.

^a^Adjusted for above covariates plus eGFR, HAb1c, TC, TG, HDL-C, and LDL-C.

^b^Adjusted for above covariates plus eGFR.

^c^Adjusted for the above covariates plus eGFR, systolic blood pressure, TC, TG, HDL-C, and LDL-C.

^d^Adjusted for above covariates plus eGFR, HAb1c, and systolic blood pressure.

^e^Adjusted for above covariates plus eGFR, HAb1c, dyslipidemia, and hypertension.

^f^Adjusted for above covariates plus HAb1c, dyslipidemia, and hypertension.

**Fig. 3:**
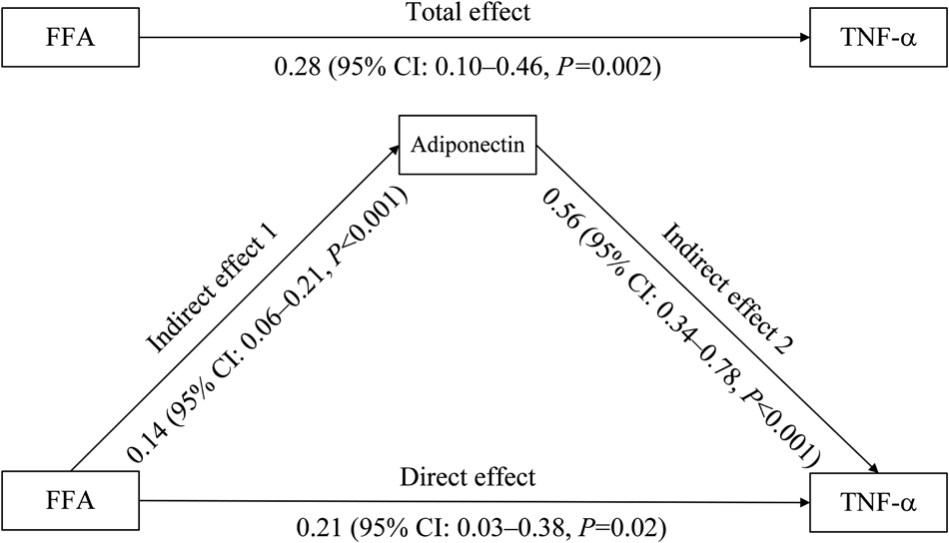
Mediating effect of adiponectin on the association between FFA and TNF-α. *FFA* free fatty acid, *TNF-α* tumor necrosis factor-α. The model had a sample size of 475 and was adjusted for age, sex, duration of diabetes, body mass index, alanine transaminase, estimated glomerular filtration rate, glycosylated hemoglobin, triglyceride, total cholesterol, high-density lipoprotein cholesterol, low-density lipoprotein cholesterol, current insulin therapy, current oral hypoglycemic agents treatment, and current statins therapy. FFA adiponectin, and TNF-a were log-transformed due to skewness distribution.

After further analyzing the association among FFA, adiponectin, and TNF-α in the mediation model, we found that FFA was consistently and positively associated with TNF-α before (total effect = 0.28, 95%CI: 0.10–0.46, *P* = 0.002) and after (direct effect = 0.21, 95%CI: 0.03–0.38, *P* = 0.02) adjustment for adiponectin. More importantly, FFA was positively associated with adiponectin (indirect effect 1 = 0.14, 95%CI: 0.06–0.21, *P* < 0.001) and adiponectin was also positively associated with TNF-α (indirect effect 2 = 0.56, 95%CI: 0.34–0.78, *P* < 0.001) (Fig. 3).

To determine whether the mediating effect of adiponectin on the association between FFA and TNF-α differed in participants with distinct characteristics, we performed subgroup analyses. We found the mediating effect of adiponectin specifically occurred in participants with younger age (percentage = 25.8%, 95%CI: 2.7–48.8, *P* = 0.03), male (percentage = 24.0%, 95%CI: 2.3–45.7, *P* = 0.03), higher HbA1c (percentage=19.4%, 95%CI: 1.8–37.1, *P* = 0.03), and in participants without current insulin therapy (percentage = 19.2%, 95%CI: 1.2–37.2, *P* = 0.04) (Supplementary Table S2).

## Discussion

In this study, we found that FFA was positively associated with adiponectin in patients with T2DM. In addition, FFA was inverted J-shaped associated with adiponectin, with adiponectin significantly increasing when FFA increased from low levels to 0.7 mmol/L but not thereafter. More importantly, we found that elevated adiponectin in turn mediated the association between FFA and TNF-α, with a 26.8% mediating effect percentage. These findings suggest that adiponectin may have pro-inflammatory effects in FFA-induced inflammation in patients with T2DM.

In this study, we found that FFA was positively associated with adiponectin in patients with T2DM and the association was robust after excluding potential bias of reverse causality. This finding was consistent with the studies conducted in the general populations of children and adults [5, 6, 20], and added novel evidence on the upregulation of adiponectin by FFA in T2DM. Adiponectin is secreted in large quantities primarily from adipose tissue and its gene expression is mainly regulated by peroxisome proliferator-activated receptor gamma (PPARγ) pathway [1]. Evidence has found that FFA can bind directly to PPARγ and increase the expression of PPARγ [21, 22], thus FFA may stimulate adiponectin secretion via PPARγ pathway activation. Nevertheless, we further found that the above positive association was only restricted to a certain range of FFA (<0.7 mmol/L) rather than to the all range. This finding could be partially explained by a limited capacity for adiponectin production in T2DM. Compared to people without diabetes, patients with diabetes are often accompanied by more risk factors, such as adiponectin gene variation, obesity, hyperglycemia, oxidative stress, and unhealthy lifestyle, all of which inhibit the expression and secretion of adiponectin [23–25]. Therefore, it is conceivable that adiponectin levels in T2DM cannot always maintain an upward trend despite the increase of FFA.

Currently, the association of adiponectin with inflammatory cytokine remains contradictory in human studies. It is accepted that adiponectin has anti-inflammatory effects in various disease states, including diabetes, nonalcoholic fatty liver disease, and cardiovascular disease [1]. In contrast, some studies support that adiponectin was positively associated with inflammatory cytokine in the condition of prediabetes, rheumatoid arthritis, Crohn disease, etc [2, 26]. In line with these studies, we found that adiponectin mediated the association between FFA and TNF-α, indicating that adiponectin might have pro-inflammatory rather than anti-inflammatory effects in T2DM. Several mechanisms may involve counterintuitively pro-inflammatory effects. First, adiponectin can induce human macrophage and T-cell differentiation to pro-inflammatory phenotype that resembles M1 other than M2 [26]. Second, adiponectin stimulates the release of TNF-α and interleukin 6 in human adipose tissues through the activation of nuclear factor-kappa B pathway and extracellularly regulated kinase pathway [1, 26]. Third, adiponectin induces the synthesis of interleukin 6 and matrix metalloproteinase inhibitor 1 in human synovial fibroblasts via a p38 mitogen-activated protein kinase pathway [2]. Although the “adiponectin paradox” in T2DM has been known for decades, the clinical data supporting the pro-inflammatory effect of adiponectin are limited in this population. The findings of this study might provide a possible explanation for this paradox from the perspective of adiponectin-promoting inflammation. However, some have proposed that under certain chronic inflammatory conditions, the pro-inflammatory effect of adiponectin might be beneficial to cells by desensitizing them to further pro-inflammatory activity [1]. Therefore, further research is required to understand the true contribution of such a pro-inflammatory effect in cardiovascular homeostasis and all-cause mortality.

In addition, we found that the mediating effect of adiponectin specifically occurred in participants with younger age, male, higher HbA1c, as well as in participants without current insulin therapy. To explain these results, we further analyzed the indirect effects of FFA on adiponectin and adiponectin on TNF-α. We found that adiponectin was consistently and positively associated with TNF-α regardless of participant characteristics (Supplementary Table S2), which still supports the potential pro-inflammatory effect of adiponectin in T2DM. However, there was heterogeneity in the association between FFA and adiponectin in participants with different characteristics (Supplementary Table S2). This may explain why the mediating effect of adiponectin was statistically significant in parts of participants. Additionally, it cannot rule out the influence of low test power as subgroups with no statistical differences have comparatively smaller sample sizes (Supplementary Table S2).

This study found that HDL-C and TG were associated with adiponectin. Evidence has shown that adiponectin can modulate lipid metabolism through the activation of peroxisome proliferator-activated receptor, 5’ adenosine monophosphate-activated protein kinase (AMPK), and acetyl CoA carboxylase, thereby further affecting blood lipid levels [27]. For example, AMPK is a critical metabolic sensor that regulates the energy homeostasis of cells. Elevated adiponectin levels upregulate the activation of AMPK, which promotes fatty acid oxidation and inhibits TG and cholesterol synthesis [27]. Furthermore, two studies conducted in people without diabetes have demonstrated that adiponectin levels were positively correlated with HDL-C levels but were negatively correlated with TG levels [28, 29], which were in line with our findings.

In addition, adiponectin also protects the liver from injury. Animal experiments found that the up-regulation of adiponectin induced by ginsenoside Rb1 contributes to the amelioration of liver steatosis [30]. The mice with high expression of adiponectin had lower TG accumulation in hepatocytes and lower ALT levels [30]. Similarly, the study conducted in patients with non-alcoholic steatohepatitis showed that participants treated by PPARγ agonist had less hepatic steatosis and necroinflammation partly due to elevated adiponectin levels [31]. Decrease in steatosis and disease activity score were closely associated with the increase in adiponectin levels [31]. The above studies suggest that elevated adiponectin may be a protective factor for the liver and liver function. Thus, it is conceivable that there should be a negative association between adiponectin and ALT as ALT is a biomarker of liver injury.

This study has several strengths. First, we eliminated the reverse causality of adiponectin on FFA and confirmed a robust result. Second, we treated adiponectin as a mediator and analyzed its mediating effect on the association between FFA and TNF-α, which can provide a deeper insight into the biological role of adiponectin in a specific pathological pathway. Nevertheless, there remain several limitations. First, causal inference should be cautious because of the cross-sectional design. Second, this study only included participants with T2DM. More studies are needed to verify the pro-inflammatory of adiponectin in other types of diabetes and chronic conditions. Third, due to data availability, we only used TNF-α to represent the inflammatory cytokine. It is warranted more studies to investigate the association between adiponectin and other inflammatory cytokines.

In conclusion, FFA was positively associated with adiponectin when FFA was less than 0.7 mmol/L but not thereafter in patients with T2DM. Additionally, elevated adiponectin mediated FFA-related inflammatory reaction. This study may provide insights into the pro-inflammatory effect of adiponectin and contribute to our knowledge of the “adiponectin paradox” phenomenon in T2DM.

### Supplementary information

Supplementary Information

## Data Availability

The data underlying this article will be shared on reasonable request to the corresponding author.

## Code availability

The SAS code underlying this article will be shared on reasonable request to the corresponding author.

## References

[1] Fang H, Judd RL (2018). Adiponectin regulation and function. Compr Physiol.

[2] Menzaghi C, Trischitta V (2018). The adiponectin paradox for all-cause and cardiovascular mortality. Diabetes.

[3] Schrieks IC, Nozza A, Stahli BE, Buse JB, Henry RR, Malmberg K (2018). Adiponectin, free fatty acids, and cardiovascular outcomes in patients with type 2 diabetes and acute coronary syndrome. Diabetes Care.

[4] Bergman RN, Ader M (2000). Free fatty acids and pathogenesis of type 2 diabetes mellitus. Trends Endocrinol Metab.

[5] Bernstein EL, Koutkia P, Ljungquist K, Breu J, Canavan B, Grinspoon S (2004). Acute regulation of adiponectin by free fatty acids. Metabolism.

[6] Krzyzanowska K, Mittermayer F, Krugluger W, Roden M, Schernthaner G, Wolzt M (2007). Adiponectin concentrations increase during acute FFA elevation in humans treated with rosiglitazone. Horm Metab Res.

[7] Fayad R, Pini M, Sennello JA, Cabay RJ, Chan L, Xu A (2007). Adiponectin deficiency protects mice from chemically induced colonic inflammation. Gastroenterology.

[8] Cheng X, Folco EJ, Shimizu K, Libby P (2012). Adiponectin induces pro-inflammatory programs in human macrophages and CD4+ T cells. J Biol Chem.

[9] Müller-Ladner U, Neumann E (2009). Rheumatoid arthritis: the multifaceted role of adiponectin in inflammatory joint disease. Nat Rev Rheumatol.

[10] Yamamoto K (2005). Production of adiponectin, an anti-inflammatory protein, in mesenteric adipose tissue in Crohn’s disease. Gut.

[11] Tripathy D, Mohanty P, Dhindsa S, Syed T, Ghanim H, Aljada A (2003). Elevation of free fatty acids induces inflammation and impairs vascular reactivity in healthy subjects. Diabetes.

[12] Xu SY, Li K, Zhang Z, Liu CY, Guo QY, Lu B (2021). Association between time in range, a novel measurement of glycemic control and islet secretory function in Chinese patients with type 2 diabetes mellitus: an observational study. Diabetes Res Clin Pract.

[13] Ye J, Deng J, Liang W, Luo H, Mei W, Liu L (2022). Time in range assessed by capillary blood glucose in relation to insulin sensitivity and beta-cell function in patients with type 2 diabetes mellitus: a cross-sectional study in China. J Diabetes Investig.

[14] Levey AS, Stevens LA, Schmid CH, Zhang YL, Castro AF, Feldman HI (2009). A new equation to estimate glomerular filtration rate. Ann Intern Med.

[15] Wallace TM, Levy JC, Matthews DR (2004). Use and abuse of HOMA modeling. Diabetes Care.

[16] Chinese Diabetes Society. (2021). Guideline for the prevention and treatment of type 2 diabetes mellitus in China (2020 edition). Chin J Diabetes Mellitus.

[17] Wang Z, Chen Z, Zhang L, Wang X, Hao G, Zhang Z (2018). Status of hypertension in China: results from the China hypertension survey, 2012-2015. Circulation.

[18] Lu Y, Zhang H, Lu J, Ding Q, Li X, Wang X (2021). Prevalence of dyslipidemia and availability of lipid-lowering medications among primary health care settings in China. JAMA Netw Open.

[19] Levey AS, Coresh J, Balk E, Kausz AT, Levin A, Steffes MW (2003). National kidney foundation practice guidelines for chronic kidney disease: evaluation, classification, and stratification. Ann Intern Med.

[20] Sabin MA, De Hora M, Holly JMP, Hunt LP, Ford AL, Williams SR (2007). Fasting nonesterified fatty acid profiles in childhood and their relationship with adiposity, insulin sensitivity, and lipid levels. Pediatrics.

[21] Kliewer SA, Sundseth SS, Jones SA, Brown PJ, Wisely GB, Koble CS (1997). Fatty acids and eicosanoids regulate gene expression through direct interactions with peroxisome proliferator-activated receptors alpha and gamma. Proc Natl Acad Sci USA.

[22] Chen Y, Li Y, Wang Y, Wen Y, Sun C (2009). Berberine improves free-fatty-acid–induced insulin resistance in l6 myotubes through inhibiting peroxisome proliferator–activated receptor γ and fatty acid transferase expressions. Metabolism.

[23] Fisman EZ, Tenenbaum A (2014). Adiponectin: a manifold therapeutic target for metabolic syndrome, diabetes, and coronary disease?. Cardiovasc Diabetol.

[24] Purnamasari D, Khumaedi AI, Soeroso Y, Marhamah S (2019). The influence of diabetes and or periodontitis on inflammation and adiponectin level. Diabetes Metab Syndr.

[25] Kadowaki T (2006). Adiponectin and adiponectin receptors in insulin resistance, diabetes, and the metabolic syndrome. J Clin Invest.

[26] Huang K, Liang Y, Ma Y, Wu J, Luo H, Yi B (2022). The variation and correlation of serum adiponectin, nesfatin-1, IL-6, and TNF-α levels in prediabetes. Front Endocrinol.

[27] Ghadge AA, Khaire AA, Kuvalekar AA (2018). Adiponectin: a potential therapeutic target for metabolic syndrome. Cytokine Growth Factor Rev.

[28] Tschritter O, Fritsche A, Thamer C, Haap M, Shirkavand F, Rahe S (2003). Plasma adiponectin concentrations predict insulin sensitivity of both glucose and lipid metabolism. Diabetes.

[29] Cnop M, Havel PJ, Utzschneider KM, Carr DB, Sinha MK, Boyko EJ (2003). Relationship of adiponectin to body fat distribution, insulin sensitivity and plasma lipoproteins: evidence for independent roles of age and sex. Diabetologia.

[30] Li Y, Zhang S, Zhu Z, Zhou R, Xu P, Zhou L (2022). Upregulation of adiponectin by ginsenoside Rb1 contributes to amelioration of hepatic steatosis induced by high fat diet. J Ginseng Res.

[31] Gastaldelli A, Sabatini S, Carli F, Gaggini M, Bril F, Belfort-DeAguiar R (2021). Ppar-γ-induced changes in visceral fat and adiponectin levels are associated with improvement of steatohepatitis in patients with NASH. Liver Int.

