# Supplementary material for: Mediating effect of adiponectin between free fatty acid and tumor necrosis factor-α in patients with diabetes
Source: Nutr Diabetes. 2024 Jun 17;14:45. doi: 10.1038/s41387-024-00302-5 (PMC11183252; doi:10.1038/s41387-024-00302-5)
Supplement: Supplementary file 1 — Supplementary Information [file 41387_2024_302_MOESM1_ESM.pdf]

Supplementary Information

Tables

Table S1 Factors associated with adiponectin in participants with type 2 diabetes

Factor	*b*	95% CI	st *b*	*P* value
Age	-0.001	-0.007–0.006	-0.016	0.80
Male sex	-0.017	-0.148–0.115	-0.013	0.80
Duration of diabetes [a]	0.002	-0.022–0.027	0.011	0.84
Body mass index	0.003	-0.017–0.023	0.022	0.75
Triglyceride [a]	-0.144	-0.240 to -0.048	-0.167	0.003
High-density lipoprotein cholesterol [a]	0.170	0.005–0.334	0.107	0.04
Free fatty acid [a]	0.126	0.036–0.215	0.141	0.006
Fasting blood glucose	0.028	-0.006–0.062	0.094	0.11
Fasting insulin [a]	-0.052	-0.141–0.037	-0.067	0.25
Alanine transaminase [a]	-0.238	-0.404 to -0.072	-0.267	0.005
Aspartate aminotransferase [a]	0.220	-0.009–0.450	0.169	0.06
Current insulin therapy	0.053	-0.107–0.212	0.040	0.52
Current incretin glucagon-like peptide 1 treatment	-0.017	-0.190–0.156	-0.012	0.85
Current glinides treatment	0.135	-0.011–0.282	0.092	0.07
Current thiazolidinediones treatment	0.084	-0.496–0.664	0.015	0.77
Current statins therapy	0.114	-0.020–0.248	0.093	0.09

Abbreviations: st, standardization.

Total cholesterol, low-density lipoprotein cholesterol, glycosylated hemoglobin, current alpha-glucosidase inhibitor treatment, current sodium-glucose cotransporter-2 inhibitors treatment, current biguanides treatment, current sulfonylureas treatment, current dipeptidyl peptidase-4 inhibitor treatment, and current fibrates therapy did not show in the table because their *P* values were >0.10 in the univariable linear regression analysis.

The current multivariable model had a sample size of 370. Adiponectin was log-transformed due to skewness distribution.

[a] Log-transformed due to skewness distribution.

Table S2 Mediating effect of adiponectin on the association between FFA and TNF-α in participants with distinct characteristics

Subgroup	N	Effect of FFA on adiponectin		Effect of adiponectin on TNF-α		Mediating effect of adiponectin		Mediating effect percentage of adiponectin	
		Coefficient (95% CI)	*P* value	Coefficient (95% CI)	*P* value	Coefficient (95% CI)	*P* value	Coefficient (95% CI)	*P* value
Age [a]									
<50 years	258	0.18 (0.09–0.26)	<0.001	0.62 (0.28–0.96)	<0.001	0.11 (0.03–0.19)	0.009	25.8 (2.7–48.8)	0.03
≥50 years	217	0.12 (0.00–0.23)	0.05	0.47 (0.18–0.76)	0.002	0.05 (-0.01–0.12)	0.09	39.9 (-41.5–121.2)	0.34
Sex [b]									
Female	156	-0.02 (-0.14–0.10)	0.71	0.68 (0.33–1.04)	<0.001	-0.02 (-0.10–0.07)	0.71	-14.5 (-108.8–79.8)	0.76
Male	319	0.22 (0.13–0.31)	<0.001	0.45 (0.16–0.74)	0.003	0.10 (0.02–0.17)	0.01	24.0 (2.3–45.7)	0.03
HbA1c [c]									
<9.0%	222	0.11 (-0.01–0.22)	0.07	0.58 (0.28–0.88)	<0.001	0.06 (-0.01–0.14)	0.10	54.8 (-80.9–190.5)	0.43
≥9.0%	253	0.17 (0.08–0.26)	<0.001	0.53 (0.19–0.86)	0.002	0.09 (0.02–0.16)	0.02	19.4 (1.8–37.1)	0.03
Current insulin therapy [d]									
No	346	0.20 (0.12–0.27)	<0.001	0.40 (0.11–0.69)	0.006	0.08 (0.01–0.14)	0.02	19.2 (1.2–37.2)	0.04
Yes	129	0.01 (-0.15–0.17)	0.91	0.81 (0.48–1.14)	<0.001	0.01 (-0.12–0.14)	0.91	20.0 (-301.5–341.4)	0.90

Abbreviations: FFA, free fatty acid; TNF-α, tumor necrosis factor-α; HbA1c, glycosylated hemoglobin.
Mediating effect of adiponectin was equal to the effect of FFA on adiponectin multiplied by the effect of adiponectin on TNF-α.
FFA, TNF-α, and adiponectin were log-transformed due to skewness distribution.
Covariates included age, sex, duration of diabetes, body mass index, alanine transaminase, estimated glomerular filtration rate, HbA1c, triglyceride, total cholesterol, high-density lipoprotein cholesterol, low-density lipoprotein cholesterol, current insulin therapy, current oral hypoglycemic agents treatment, and current statins therapy.
[a] Adjusted for above covariates except for age.
[b] Adjusted for above covariates except for sex.
[c] Adjusted for above covariates except for HbA1c.
[d] Adjusted for above covariates except for current insulin therapy.
